# *Ex vivo*-expanded highly purified natural killer cells in combination with temozolomide induce antitumor effects in human glioblastoma cells *in vitro*

**DOI:** 10.1371/journal.pone.0212455

**Published:** 2019-03-06

**Authors:** Yoshitaka Tanaka, Tsutomu Nakazawa, Mitsutoshi Nakamura, Fumihiko Nishimura, Ryosuke Matsuda, Koji Omoto, Yoichi Shida, Toshiharu Murakami, Ichiro Nakagawa, Yasushi Motoyama, Hiromichi Morita, Takahiro Tsujimura, Hiroyuki Nakase

**Affiliations:** 1 Department of Neurosurgery, Nara Medical University, Kashihara, Nara, Japan; 2 Grandsoul Research Institute for Immunology, Inc., Uda, Nara, Japan; 3 Clinic Grandsoul Nara, Uda, Nara, Japan; Sechenov First Medical University, RUSSIAN FEDERATION

## Abstract

Glioblastoma is the leading malignant glioma with a poor prognosis. This study aimed to investigate the antitumor effects of natural killer cells in combination with temozolomide as the standard chemotherapeutic agent for glioblastoma. Using a simple, feeder-less, and chemically defined culture method, we expanded human peripheral blood mononuclear cells and assessed the receptor expression, natural killer cell activity, and regulatory T cell frequency in expanded cells. Next, using the standard human glioblastoma cell lines (temozolomide-sensitive U87MG, temozolomide-resistant T98G, and LN-18), we assessed the ligand expressions of receptors on natural killer cells. Furthermore, the antitumor effects of the combination of the expanded natural killer cells and temozolomide were assessed using growth inhibition assays, apoptosis detection assays, and senescence-associated β-galactosidase activity assays in the glioblastoma cell lines. Novel culture systems were sufficient to attain highly purified (>98%), expanded (>440-fold) CD3^−^/CD56^+^ peripheral blood-derived natural killer cells. We designated the expanded population as genuine induced natural killer cells. Genuine induced natural killer cells exhibited a high natural killer activity and low regulatory T cell frequency compared with lymphokine-activated killer cells. Growth inhibition assays revealed that genuine induced natural killer cells inhibited the glioblastoma cell line growth but enhanced temozolomide-induced inhibition effects in U87MG. Apoptosis detection assays revealed that genuine induced natural killer cells induced apoptosis in the glioblastoma cell lines. Furthermore, senescence-associated β-galactosidase activity assays revealed that temozolomide induced senescence in U87MG. Genuine induced natural killer cells induce apoptosis in temozolomide-sensitive and temozolomide-resistant glioblastoma cells and enhances temozolomide-induced antitumor effects in different mechanisms. Hence, the combination of genuine induced natural killer cells and temozolomide may prove to be a promising immunochemotherapeutic approach in patients with glioblastoma if the antitumor effects *in vivo* can be demonstrated.

## Introduction

Glioblastoma (GBM) is the most lethal malignant tumor of the brain. The current standard therapy combines maximal surgical tumor resection with adjuvant therapy, comprising temozolomide (TMZ) chemotherapy, and multifractionated radiation (total dose: 60 Gy) [1]. Although this therapy shows improved outcomes, the overall 5-year survival rate [9.8% with TMZ vs. 1.9% (0.6%–4.4%) with radiotherapy alone (hazard ratio, 0.6; 95% confidence interval: 0.5–0.7; P < 0.0001)] in patients with GBM remains poor [2], necessitating the implementation of more novel and effective treatment strategies.

Natural killer (NK) cells, defined as the absence of CD3 and presence of CD56, constitute approximately 10% of all lymphocytes in the human peripheral blood [3]. NK cells exhibit potent cytotoxic activity against tumor cells *via* apoptosis [4] and can remove abnormal cells including tumor and virus-infected cells as the innate immune system [5,6]. These cells recognize tumor cells by forming a synapse with the tumor cells and induce apoptosis by releasing cytotoxic molecules such as perforin and granzyme against the tumor cells [7]. Perforin forms pores on the tumor to deliver granzymes into the tumor cells [8], and granzyme-activated caspase induces tumor cell apoptosis [9]. The cytotoxic function of NK cells is ascertained by the balance between activating and inhibitory receptor signals [10,11]. Some ligands binding to the activating receptors of NK cells, such as NKG2D and DNAM-1, are expressed in GBM [12], and the ligation of the activating receptors triggers cytotoxicity in NK cells [13]. Ligands of NK inhibitory receptors, such as NKG2A and KIR2DL, are also associated with NK cell cytotoxicity against tumor cells [14,15].

Multiple clinical studies on various tumors have validated NK cells as a promising therapeutic option for treating malignant tumors [16,17]. Since the late 1980s, the efficacy of adoptively transferred autologous lymphokine-activated killer cells (LAK) has been investigated comprehensively [18]. Treatment with intralesional autologous LAK was reportedly safe and exhibited extended survival [19]. However, clinical applications of NK cells, especially to GBM, have been scarcely reported because of difficulty in the large-scale expansion and production of highly purified NK cells [20]. Furthermore, the T-cell component of LAK can inhibit the NK activity because of the development of regulatory T cells (Tregs) [21].

This study aimed to (a) develop highly purified human NK cells with robust cytotoxic activity derived from peripheral blood mononuclear cells (PBMCs) using a simple, feeder-less method, such as cancer cells; (b) investigate the cellular characteristics of NK cells, including receptor expression, NK activity, and frequency of Tregs in the expanded populations; and (c) investigate the antitumor effects of the expanded NK cells in combination with TMZ, which is the standard chemotherapy agent for GBM, and the mechanisms of the cytotoxicity against GBM *in vitro*. We designated the expanded NK cells using the novel culture system as genuine induced NK cells (GiNK).

## Materials and methods

### Reagents

TMZ was purchased from MSD Inc. (Tokyo, Japan).

### Cell lines

This study was approved by Nara medical university ethics committee. The approval number is 1058. We obtained standard human GBM cell lines—U87MG, T98G, and LN-18—from the American Type Culture Collection (ATCC; Manassas, VA). Human leukemia cell line K562 was provided by Professor Osamu Mazda (Kyoto Prefectural University of Medicine, Kyoto, Japan). We maintained GBM cells in Dulbecco’s modified Eagle’s medium (DMEM; Life Technologies, Carlsbad, CA) and K562 cells in Roswell Park Memorial Institute-1640 medium (Life Technologies) supplemented with 10% heat-inactivated fetal bovine serum (FBS; MP Biomedicals, Tokyo, Japan), 100 U/mL penicillin, and 100 μg/mL streptomycin (Life Technologies) at 37°C in a humidified 5% CO_2_-containing atmosphere.

### *Ex vivo* expansion of human genuine induced NK cells

We prepared PBMCs from 8 ml of heparinized peripheral blood obtained from healthy volunteers (mean age, 33.5 years) using a conventional preparation kit (Lymphoprep™; Axis-Shield PoC AS, Oslo, Norway) as per manufacturer’s instructions. The PBMCs were depleted in the CD3 fraction by the RosetteSep™ Human CD3 Depletion Cocktail (STEMCELL Technologies, Vancouver, Canada). We placed the CD3-depleted PBMCs in a T25 culture flask (Corning, Steuben, NY) containing AIM-V medium (Life Technologies) at 37°C in a humidified 5% CO_2_-containing atmosphere, supplemented with 5% autologous plasma, IL-18 (Medical & Biological Laboratories Co., Ltd.; MBL, Nagoya, Japan), and 3000 IU/mL recombinant human (rh) interleukin-2 (IL-2; Novartis, Basel, Switzerland) for 14 days. All procedures performed in studies involving human participants were in accordance with the ethical standards of the institutional and/or national research committee and with the 1964 Helsinki declaration and its later amendments or comparable ethical standards. Informed consent was obtained from all healthy volunteers included in the study.

### Cytotoxicity assays

We measured the cytotoxic effect using the calcein-AM release assay, as described previously [22], to assess differences in the NK activity between GiNK and LAK. Briefly, we incubated NK activity-sensitive leukemia cell line K562, used as a target with 1 μM calcein-AM (Life Technologies), for 30 min and then washed it twice with 5% FBS containing Hanks’ balanced salt solution. Then, GiNK or LAK used as effectors were added to 96-well round-bottomed plastic plates (Corning) containing 10^4^ calcein-AM–labeled K562 per well at effector-to-target (E:T) ratios of 1:5. After 4-h incubation at 37°C in a humidified atmosphere, we collected the supernatants and measured calcein-AM release using Fluoroskan Ascent (Thermo Fisher Scientific).

### Expression analysis of cell surface antigens

We stained cells with appropriate antibodies, which were analyzed using a BD FACSCalibur flow cytometer (BD Biosciences, Franklin Lakes, NJ). Data were analyzed using CellQuest software ver 6.0 (BD Biosciences). We stained GBM cell lines using the following mouse anti-human antibodies: anti-CD112-PE (R2.525) and anti-CD50-PE (TU41)—BD Biosciences; anti-MIC-A-PE (AMO1) and anti-MIC-B-PE (BMO1)—MBL; anti-ULBP-1-PE (170818), anti-ULBP-2-PE (165903), and anti-ULBP-3-PE (166510)—R&D Systems (Minneapolis, MN); and anti-HLA-ABC-PE (W6/32), anti-HLA-E-PE (3D12), anti-CD48-PE (156-4H9), anti-CD155-PE (2H7CD155), anti-CD54-PE (HA58), and anti-CD102-PE (CBRIC2/2)—Thermo Fisher Scientific (Waltham, MA).

The expanded NK cells were stained with the following antibodies: anti-CD3-FITC (UCHT1), anti-CD56-PE-Cy5 (B159), anti-CD11a-PE (HI111), anti-CD16-PE (3G8), anti-DNAM-1-PE (DX11), anti-NKp30-PE (P30-15), anti-NKp44-PE (P44-8.1), anti-NKp46-PE (9E2/NKp46), and anti-CD161-PE (NKR-P1A)—BD Biosciences; anti-CD158a-PE (EB6Bf) and anti-CD159a-PE (NKG2A)—Beckman Coulter (Pasadena, CA); anti-NKG2D-PE (149810)—R&D Systems; anti-CD2-PE (RPA-2.10), anti-CD4-PE (OKT4), anti-CD8-PE (SK1), anti-CD244-PE (C1.7), anti-CD94-FITC (DX22), and anti-CD158b-PE (GL183)—Thermo Fisher Scientific.

### Determination of Treg frequency

We stained the expanded cells with the Anti-Human Foxp3 PE Staining Set (BD Biosciences) and anti-CD4-PE (OKT4) per the manufacturer’s instructions to evaluate the Treg frequency in the expanded PBMCs, including GiNK and LAK. The stained cells were analyzed using a FACSCalibur flow cytometer and CellQuest Pro software ver 6.0.

### Growth inhibition assays

We seeded GBM cell lines in 24-well flat-bottomed plastic plates at 2 × 10^4^ cells/well in 0.4 mL DMEM supplemented with 10% heat-inactivated FBS, 100 U/mL penicillin, and 100 μg/mL streptomycin (Life Technologies). After attaching target cells (GBM cell lines) to the 24-well flat-bottomed plastic plates (Corning), these were co-incubated with GiNK at various E:T ratios of 0:1, 1:1, and 2:1 in the presence/absence of 50 μM TMZ. Next, we incubated the plates for 96 h at 37°C in a humidified 5% CO_2_-containing atmosphere. We used phosphate-buffered saline (PBS; COSMO Bio, Tokyo, Japan) and AIM-V medium as a control for TMZ and GiNK, respectively. Following incubation, while we discarded any nonadherent cells, adherent cells were trypsinized and stained with trypan blue dye. Then, we calculated the number of viable non-stained cells using manual counting and Countess™ Automated Cell Counter (Life Technologies). Relative cell numbers (%) were evaluated using the following formula: (viable cell numbers in the presence of TMZ and/or GiNK cells) / (viable cell numbers in the presence of PBS) × 100.

### Apoptosis detection assays

We performed apoptosis detection assays using the MEBCYTOTM Apoptosis Kit (MBL), per the manufacturer’s instructions. Briefly, GBM cell lines were exposed to GiNK at E:T ratios of 1:1 in the presence/absence of 50μM TMZ for 24 h. The E:T ratio (1:2) of LN-18 was set as half that of other cell lines, as LN-18 was more sensitive for the NK activity than other GBM cell lines.

Following incubation, we washed floated cells and trypsinized adherent cells with PBS once, stained them with Annexin V–FITC and propidium iodide (PI), and maintained them at room temperature for 15 min in the dark. Then, we analyzed the stained cells using a FACSCalibur flow cytometer and CellQuest Pro software ver 6.0. Notably, NK cells were excluded by electronic gating based on forward-scatter and side-scatter characteristics. The frequency of the Annexin V-positive and PI-negative population was defined as apoptotic cells, as described previously [23,24].

### Senescence-associated β-galactosidase activity assays

We measured the senescence-associated β-galactosidase (SA-β-gal) activity using a β-gal staining kit (Senescence Detection Kit; BioVision Research Products, Milpitas, CA) per the manufacturer’s instructions. GBM cell line (U87MG) was exposed to NK cells at E:T ratios of 1:1 in the presence/absence of 50 μM TMZ for 96 h in a 12-well flat-bottomed plate. Then, the culture medium was discarded, and the cells were washed once with PBS, followed by fixing the cells with 0.5 mL of a fixative solution for 10–15 min at room temperature. Next, the cells were washed twice with PBS. We added a Staining Solution Mix to each well and incubated the mixture overnight for 24 h at 37°C in a humidified 5% CO_2_-containing atmosphere. Finally, cells were observed under the microscope for the development of a blue color and then counted.

### Statistical analysis

Data are presented as mean ± standard error. We determined if differences were statistically significant using a t-test, Mann–Whitney U test, and one-way analysis of variance (ANOVA) followed by Tukey’s test or Kruskal–Wallis test in conjunction with Steel–Dwass’s test. Statistical analyses were performed by BellCurve for Excel (Social Survey Research Information Co., Ltd., Tokyo, Japan). We considered P < 0.05 as statistically significant.

## Results

### Expansion of human PBMC-derived NK cells *ex vivo*

**Fig 1:**
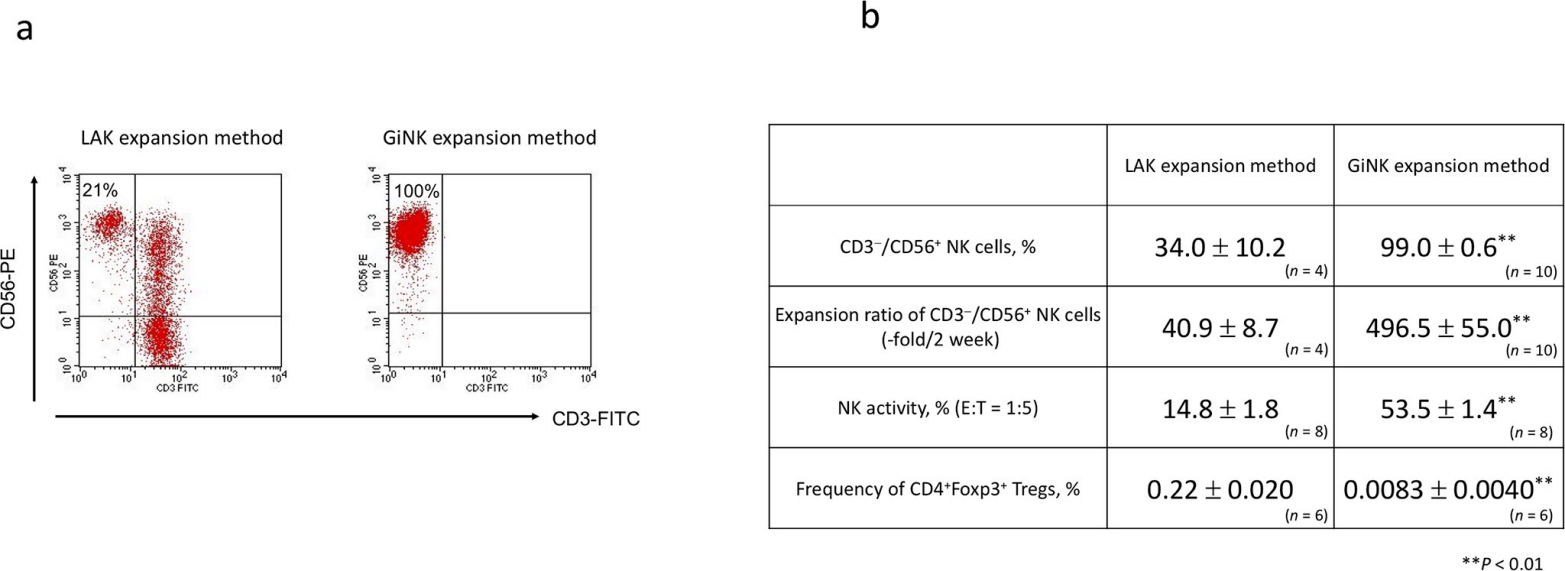
Characteristics of expanded cells from human PBMCs in different culture methods. (a) Representative flow cytometric figures depicting the frequency of CD3−CD56+ NK cells in expanded cells. The cells were stained with FITC-conjugated anti-CD3 and PE-conjugated anti-CD56. (b) The profile of cells expanded by two different culture methods. The figure shows the purity of NK cells (%), the expansion rate of NK cells (fold), the NK activity (%), and the frequency of Tregs (CD4^+^Foxp3^+^ cells, %) in two culture methods. Data indicated as mean ± SE. P values were determined using the Mann–Whitney U test to compare NK cells expanded by the newly established method and LAK expansion method. Statistically significant differences: **P < 0.01.

The newly established PBMC-derived NK cell expansion method induced highly purified CD3^−^CD56^+^ NK cells (99.0% ± 0.6%) with a high expansion rate (496.5-fold ± 55.0-fold) within 2 weeks. The LAK expansion method induced low CD3^−^CD56^+^ NK cell positivity (34.0% ± 10.2%) with a low expansion ratio (40.9-fold ± 8.7-fold) within 2 weeks. The findings indicated the superiority of the newly established NK cell culture method over the LAK expansion method (Fig 1A and 1B). We designated the expanded highly purified NK cell population as GiNK.

### Cellular characteristics of GiNK

In NK activity assays, GiNK killed 53.5%±1.4% of K562 cells; however, LAK killed 14.8% ± 1.8% at E:T ratios of 1:5. Thus, GiNK exhibited more vigorous NK activity than LAK (Fig 1B). Furthermore, the Treg frequency was significantly lower in GiNK within 2 weeks compared with LAK (0.0083% ± 0.0040% vs. 0.22% ± 0.020%; *P* < 0.01; Fig 1B).

**Fig 2:**
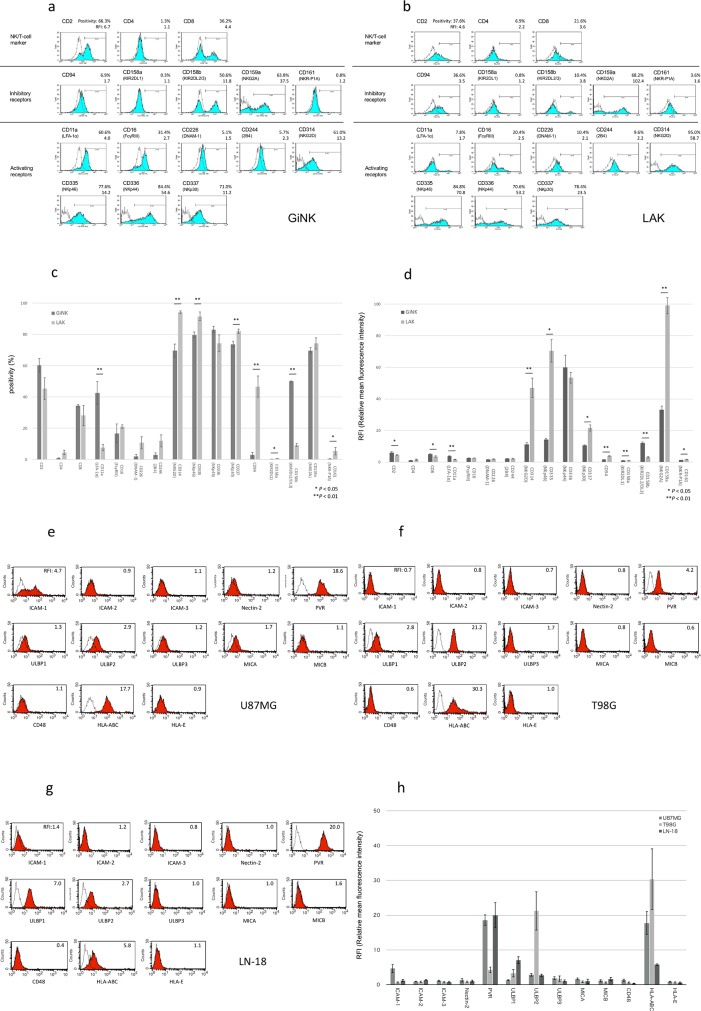
Analysis of the surface receptors’ expression on GiNK and ligands’ expression on GBM cell lines specific for receptors expressed on GiNK. (a, b) Representative data of receptor expression on GiNK (a) and LAK (b). Cells were stained with specific mAbs and analyzed using flow cytometry. Open dot line histograms, with controls stained with isotype mAb; filled histograms, specific mAb staining. Top, middle, and bottom panels show representative NK/T-cell markers, activating NK receptors, and inhibitory NK receptors, respectively. The positivity indicates the percentage of positive cells. The relative mean fluorescent intensity (RFI) divided by the overall mean fluorescence intensity of each sample from the isotype control. (c) The positivity of the surface receptor expressing cells in GiNK and LAK. The numbers given are the average of positivity obtained from three independent experiments. P values were determined using a t-test or Mann–Whitney U test: *P < 0.05, **P < 0.01. (d) The RFI of the surface receptor expression on GiNK and LAK. The numbers given are the average RFI of values obtained from three independent experiments. P values were determined using a t-test or Mann–Whitney U test: *P < 0.05, **P < 0.01. (e, f, g) Representative data of ligands expressed on GBM cell lines U87MG (e), T98G (f), LN-18 (g) specific to the receptors on NK cells. Open dot line histograms, controls stained with isotype mAb; filled histograms, specific mAb staining. The RFI divided by the overall mean fluorescence intensity of the individual sample from isotype-matched control. (h) The RFI of ligands expressed on GBM cell lines specific to the receptors on GiNK. Each bar shows the mean ± SE of values obtained from triplicate experiments.

The analysis of the expression of surface receptors revealed that GiNK expressed lineage markers CD8 and CD4, although CD4 had deficient levels. The cells expressed adhesion molecules CD2 (LFA-2) and CD11a (LFA-1a) at low levels. Of the NK cell markers, CD161 (NKR-P1A) was slightly expressed. Of the markers associated with activating NK cell receptors, CD314 (NKG2D), CD335 (NKp46), CD336 (NKp44), and CD337 (NKp30) were highly expressed, whereas CD16 (FcγRIII), CD226 (DNAM-1), and CD244 (2B4) were marginally expressed. Of the markers associated with inhibitory NK cell receptors, CD158b (KIR2DL2/DL3) and CD159a (NKG2A) were highly expressed, whereas CD94, CD158a (NIR2DL1), and CD161 (NKR-P1A) were expressed at low levels. Besides, GiNK expressed activating and inhibitory NK cell receptors, which are generally expressed in human NK cells (Fig 2A and 2C) [25]. GiNK had significantly higher expressions of CD11a and CD158b but lower expressions of CD158a, CD314, CD335, CD94, and CD161 compared with LAK in both positivity and RFI. Additionally, GiNK had significantly higher expressions of CD2 and CD8 but lower expressions of CD159a compared with LAK in RFI only (Fig 2A, 2B, 2C and 2D).

### Surface ligands’ expression on GBM cell lines specific for receptors expressed on GiNK

Regarding ligands corresponding to activating NK receptors, GBM expressed ICAM-2, ICAM-3 (ligand for LFA-1), PVR (ligand for DNAM-1), ULBP-3, MIC-A, and MIC-B (ligand for NKG2D) at low levels; however, ICAM-1 (LFA-1) was highly expressed on U87MG. While PVR was highly expressed in U87MG and LN-18, ULBP-1 (ligand for NKG2D) and ULBP-2 (ligand for NKG2D) were highly expressed in LN-18 and T98G, respectively. Regarding ligands corresponding to the inhibitory NK receptors, GBM expressed CD48 (ligand for 2B4) and HLA-E (ligand for CD94/NKG2A) at low levels and HLA-class I (ligand for KIR2DL) at high levels in GBM cell lines (Fig 2E, 2F, 2G and 2H).

### Growth inhibition effects of GiNK and TMZ on GBM cell lines *in vitro*

**Fig 3:**
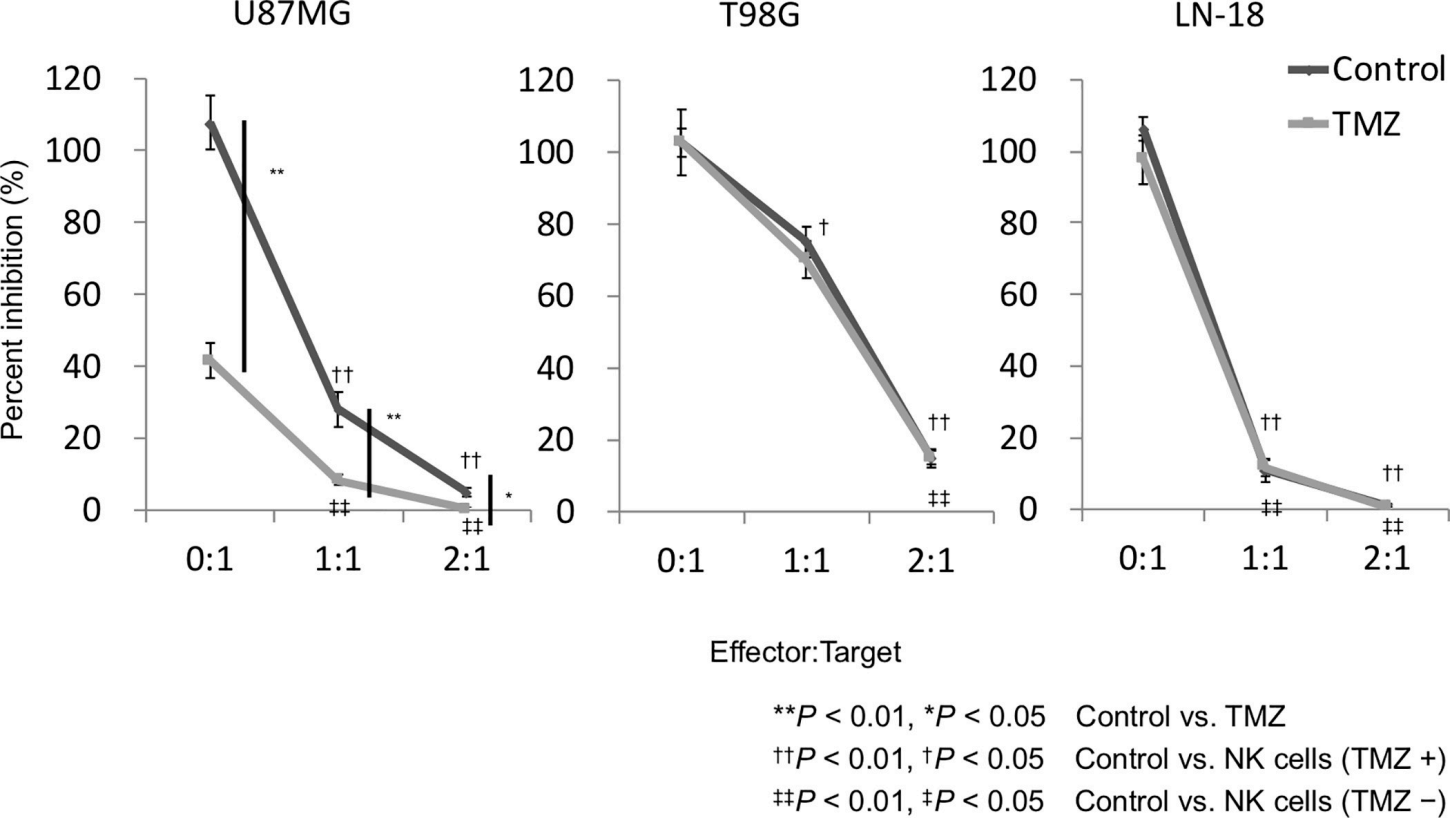
Growth inhibition effects of GiNK and TMZ on GBM cell lines *in vitro*. GBM cell lines were seeded at 2×10^4^ cells/well in 24-well flat-bottomed plates in the presence of GiNK at a GiNK:GBM cell ratio of 0:1, 1:1, or 2:1 and cultured for 96 h. Diamond and square marks, the absence and presence of 50 μM TMZ, respectively. Adherent cells were detached and determined by trypan blue exclusion. Non-stained cells were counted as viable cells. Left, center, and right graphs, U87MG, T98G, and LN-18 cells, respectively. Data indicated as mean ± SE of nine independent experiments. *P* values were determined using the Kruskal–Wallis test followed by Steel–Dwass’s test. Statistically significant differences: **P < 0.01, *P < 0.05, control versus TMZ; ^††^*P* < 0.01, ^†^*P* < 0.05, control versus NK cells (in the presence of TMZ); ^‡‡^*P* < 0.01, ^‡^*P* < 0.05, control versus NK cells (in the absence of TMZ).

In the presence of GiNK at E:T ratios of 0:1, 1:1, and 2:1 and in the absence of TMZ, the relative cell numbers of U87MG were 107.8% ± 7.5%, 28.0% ± 4.9%, and 5.0% ± 1.2%, respectively. In the presence of GiNK at E:T ratios of 0:1, 1:1, and 2:1 and in the absence of TMZ, the relative cell numbers of T98G were 102.6% ± 3.9%, 75.0% ± 4.3%, and 14.8% ± 2.6%, respectively. In the presence of NK cells at E:T ratios of 0:1, 1:1, and 2:1 and in the absence of TMZ, the relative cell numbers of LN-18 were 106.3% ± 3.3%, 10.7% ± 3.1%, and 0.9% ± 0.6%, respectively. Thus, per the E:T ratio, the relative cell numbers markedly decreased in the three cell lines without TMZ. In the presence of GiNK at E:T ratios of 0:1, 1:1, and 2:1 and in the presence of TMZ, the relative cell numbers of U87MG were 41.6% ± 4.9%, 8.5% ± 1.4%, and 0.4% ± 0.3%, respectively. In the presence of GiNK at E:T ratios of 0:1, 1:1, and 2:1 and in the presence of TMZ, the relative cell numbers of T98G were 102.7% ± 9.2%, 70.1% ± 5.2%, and 15.0% ± 1.9%, respectively. In the presence of GiNK at E:T ratios of 1:0, 1:1, and 2:1 and in the presence of TMZ, the relative cell numbers of LN-18 were 97.7% ± 6.8%, 11.7% ± 2.4%, and 0.5% ± 0.2%, respectively. Hence, per the E:T ratio, the relative cell numbers markedly declined in all cell lines in the presence of TMZ. Furthermore, GiNK enhanced TMZ-induced growth inhibition on U87MG cell lines only (Fig 3).

### Apoptosis-inducing effects of GiNK on GBM cell lines *in vitro*

**Fig 4:**
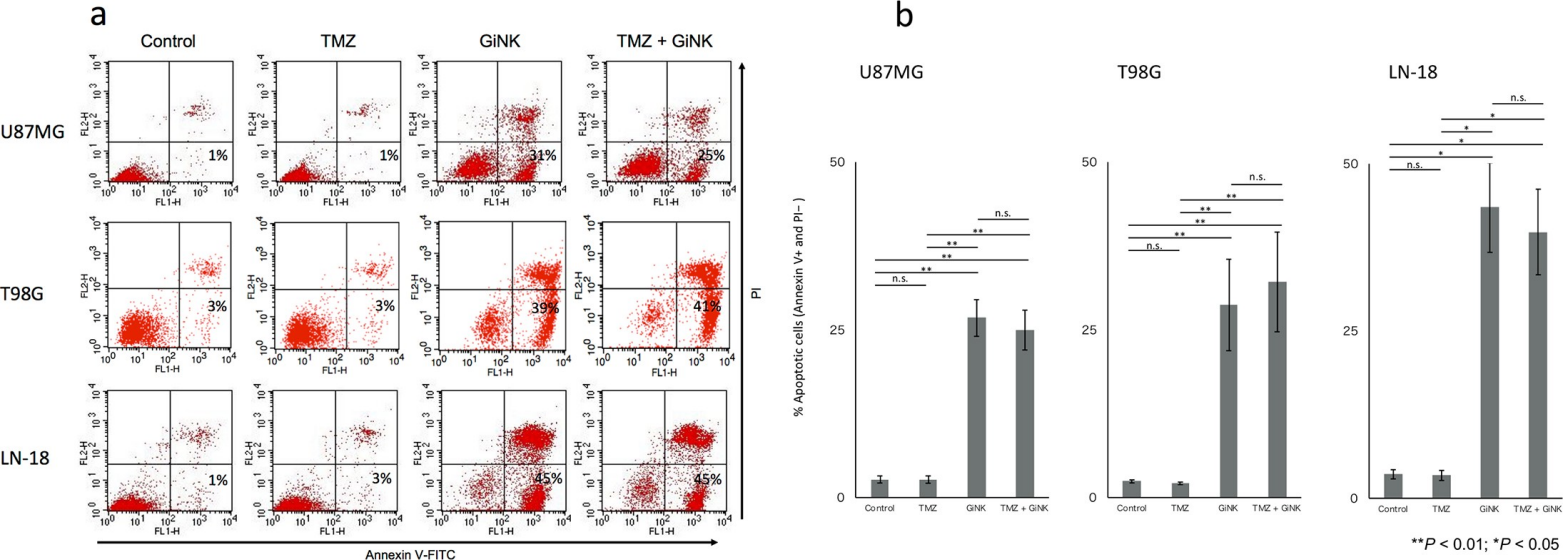
Apoptosis-inducing effects of GiNK on GBM cell lines *in vitro*. U87MG and T98G were exposed to GiNK at a GiNK:GBM cell ratio of 1:1, and LN-18 was exposed to GiNK at a GiNK:GBM cell ratio of 1:2 in the presence/absence of 50 μM TMZ for 24 h. Nonadherent and detached adherent cells were stained with Annexin V–FITC and PI, and the stained cells were analyzed using flow cytometry. Annexin V-positive and PI-negative apoptotic cells are shown as a percentage of apoptotic cells from the total number of counted cells. (a) Representative dot plots of Annexin V–PI two-color flow cytometric analysis of U87MG, T98G, and LN-18 cells, respectively. (b) The graphs show the percentages of Annexin V-positive and PI-negative apoptotic cells of U87MG (*n* = 7), T98G (*n* = 11), and LN-18 (*n* = 6) cells. Data indicated as mean ± SE. *P* values were determined using the Kruskal–Wallis test followed by Steel–Dwass’s test. Statistically significant differences: **P < 0.01, *P < 0.05.

The apoptosis detection assays revealed that after 24 h exposure, GiNK cells markedly induced apoptosis in U87MG, T98G, and LN-18 but TMZ did not induce apoptosis in all cell lines. Although GiNK/TMZ markedly induced apoptosis in GBM cell lines after 24-h exposure, they displayed no substantial additive effect compared with the GiNK administration (Fig 4A and 4B).

### Senescence-inducing effects of TMZ on GBM cell lines *in vitro*

**Fig 5:**
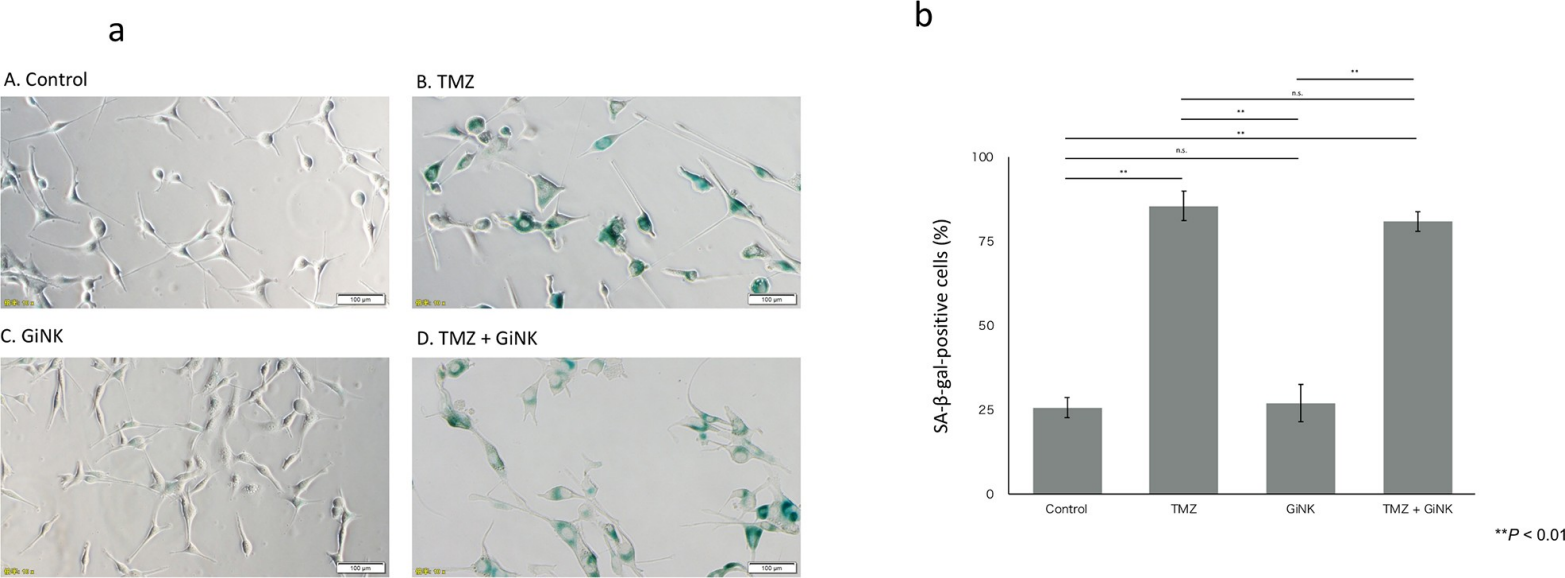
Senescence-inducing effects of TMZ on GBM cell lines *in vitro*. U87MG cells were seeded at 1 × 10^4^ cells/well in 12-well flat-bottomed plates. U87MG was exposed to GiNK at a GiNK:GBM cell ratio of 1:1 in the presence/absence of 50 μM TMZ for 96 h. Adherent cells were stained with SA-β-gal, and the stained cells were counted under an inverted microscope. (a) Representative photographs of SA-β-gal-stained U87MG cells under each treatment condition. (b) The graphs show the percentages of SA-β-gal-positive cells of U87MG (*n* = 4) cells. Data indicated as mean ± SE. *P* values were determined using one-way ANOVA followed by Tukey’s test. Statistically significant differences: **P < 0.01.

Additive effect of TMZ was observed only in U87MG in the growth inhibition assays and not observed in the apoptosis detection assays. Therefore, we investigated whether senescence, which involves a mechanism for cell growth inhibition different from that for apoptosis, occurs with a combined effect for U87MG.The SA-β-gal activity assays revealed that TMZ markedly induced senescence in U87MG, whereas GiNK did not induce senescence. Although GiNK/TMZ induced senescence in U87MG after 96-h exposure, it exhibited no marked additive effect compared with the TMZ administration (Fig 5A and 5B).

## Discussion

This study postulated a novel large-scale expansion method of NK cells using human PBMCs and a chemical defined cocktail in a relatively short period without feeder cells, including cancer and immune cells. The expanded cellular populations exhibited minimal immunosuppressive cells (Tregs), designated as GiNK. In this study, GiNK exhibited potent growth inhibitory effects in three GBM cell lines and the effects varied with the kind of GBM cell line. Furthermore, the growth inhibitory effects displayed an additional antitumor effect by the TMZ. Notably, the mechanisms underlying these growth inhibitory effects of GiNK in GBM cell lines are associated with those underlying apoptosis.

NK cells frequently infiltrate GBM and exist as one of the least numerous immune cell populations of all tumor-infiltrating immune cells in GBM [26,27]. The potential of NK cells, as effectors against brain tumors, has been established *in vitro* and *in vivo* [28]. Immunotherapies could reportedly be a promising method for GBM treatment [29]. The clinical use warrants a high-yield and high-purity method to produce activated NK cells to induce the treatment efficacy. Granzin et al. reported a large-scale expansion (390–1185 fold) method of NK cells using an irradiated Epstein–Barr virus–transformed lymphoblastoid cell line as feeder cells [30]. Lee et al. demonstrated a larger-scale (>500-fold) and a highly purified (>98%) expansion method within 2 weeks using irradiated autologous PBMCs and anti-CD16 mAb [31]. Sakamoto et al. developed a large-scale (4720-fold) and highly purified (>90%) expansion method for approximately 3 weeks, which used retronectin-stimulated T (RN-T) cells as feeder cells, requiring a relatively complicated procedure and a longer-term (4–5 weeks) culture [32]. Koehl et al. reported a feeder-free and simple method to expand NK cells from magnetic bead–selected CD3^−^/CD56^+^ cells [33]; NK cells expanded using their method were highly purified (>95%) but not attained on a large scale (five-fold) for clinical use. Conversely, we established a simple *ex vivo* culture method for GiNK, highly purified human NK cells, comprising a feeder-free and chemically defined culture system. This study obtained an approximately 500-fold expansion of lymphocytes containing about 99% expanded NK cells within 2 weeks. To the best of our knowledge, our method of selective expansion of autologous human NK cells has the highest purity and largest expansion scale with the easy and feeder-free method and can decrease the risk of bacterial and viral contamination derived from feeder cells and provide a stable amplification efficiency compared with utilizing feeder cells. Moreover, we expanded NK cells from CD3^+^ T cells–depleted PBMCs, which not only enhanced the purity of NK cells but also prevented contamination with Tregs. Reportedly, the reduction of the function and number of Tregs is beneficial for immunotherapy against malignant tumors [34]. Based on our findings, immunotherapy using GiNK might be a promising novel treatment option for GBM.

Traditional NK cells exhibit cytotoxicity in tumor cells [35]. Cytotoxicity is based on an interplay of inhibitory and activating receptor signals [36,37], and GBM cells express various molecules detected by the activating receptors on NK cells. In the growth inhibition assays, the GiNK sensitivity varied among the three cell lines. Further, the mean relative cell numbers of U87MG, T98G, and LN-18 were 28.0%, 75.0%, and 10.7%, respectively, in the presence of GiNK at E:T ratios of 1:1. Analysis of ligands expressed on the GBM cell lines revealed that T98G showed lower PVR (an NK activating receptor DNAM1 ligand) expression and growth inhibitory effect of GiNK compared with U87MG and LN-18. HLA-class I (an NK inhibitory receptor KIR2DL ligand) was highly expressed on T98G. These results show a possibility that PVR and HLA-class I are mainly associated with the differences of GiNK-induced growth inhibitory effects in the GBM cell lines we tested. From the results of the comparative analysis between GiNK and LAK, GiNK had significantly higher expressions of CD2, CD8, CD11a, and CD158b but lower expressions of CD158a, CD314, CD335, CD94, CD159a, and CD161 compared with LAK. These data indicated the possibility that GiNK could strongly recognize ICAM-1 (the specific ligand for CD11a) expressing U87MG compared with LAK and be inhibited by HLA class I-expressing U87MG, T98G, and LN-18 through CD158b. Alternatively, it is possible that compared with GiNK, LAK is activated by MIC and ULBP-expressing U87MG, T98G, and LN-18 through CD314. Furthermore, the ligand of NKp46 is still unknown in cancer cells. The GBM cell lines we tested did not express HLA-E specific ligands for CD94/CD159a. This comprehensive information suggests that LAK had a relatively strong recognition for three GBM cell lines compared with GiNK. Because it is known that NK cells recognize and respond to cancer cells by balancing many activation receptors and inhibitory receptors, including the NK receptors we tested, further detailed analyses including unknown ligand or uninvestigated receptors expression are needed.

GiNK exerts additive antitumor effects of TMZ against the TMZ-sensitive GBM cell line U87MG, and the TMZ-resistant GBM cell line LN-18 exhibited a higher growth inhibitory effect when administered with GiNK compared with other cell lines; this finding corroborates a findings of a previous study [38]. Because no correlation exists between the sensitivities of GBM of TMZ and NK cells, GiNK might be a novel therapeutic strategy for TMZ-resistant GBM treatment. This study investigated GiNK-induced apoptosis effects against TMZ-treated GBM cell lines. Reportedly, apoptosis is one of the mechanisms of NK-based immunotherapy against GBM [39]. We observed that GiNK induced apoptosis in all tested GBM cell lines, but 50 μM TMZ did not induce apoptosis. Because the additive effects of TMZ was observed only in U87MG in the growth inhibition assays but not in the apoptosis detection assays, we assessed senescence detection assay. Consequently, we found that TMZ promoted the premature senescence of U87MG and GiNK did not promote any SA-β-gal activity of U87MG. We confirmed that the additive effects of TMZ in U87MG are caused by senescence induction. Thus, the combination of TMZ and GiNK possibly additively suppressed the TMZ-sensitive GBM cells’ proliferation.

This study revealed that allogeneic activated and expanded NK cells killed GBM cells. Recently, Björklund et al. reported that highly purified haploidentical allogeneic NK cells activated by IL-2 elicited complete remission against patients with chemotherapy-refractory or relapsed high-risk myelodysplastic syndrome (MDS), secondary AML (MDS/AML), and de novo AML [40], implying that highly purified allogeneic NK cell-based immunotherapy would be safe and might provide a novel strategy for allogeneic NK cell-based immunotherapy for human GBM. Our culture systems might support these approaches when large amounts of NK cells are required.

This study has some limitations. First, we used blood from healthy volunteers. Usually, expanding NK cells from the blood of patients with cancer is challenging because of the possibility of having an immune function disorder [32]. Second, we evaluated the antitumor effect of GiNK against GBM only *in vitro*. Perhaps, adoptively transferred GiNK exhibit limited persistence; GiNK do not infiltrate the tumor or tumors develop mechanisms to evade NK cell surveillance *in vivo* [41]. Hence, our findings warrant validation in *in vivo* studies.

## Conclusions

We designated the expanded human peripheral blood NK cells using the novel culture system for clinical application as GiNK. GiNK induces apoptosis in TMZ-sensitive and TMZ-resistant GBM cells and enhances TMZ-induced antitumor effects in different mechanisms. Hence, the combination of GiNK and TMZ might be a promising immunochemotherapeutic approach in patients with GBM.

## Supporting information

S1 Dataset (XLSX)

## References

[1] Stupp R, Mason WP, Van Den Bent MJ, Weller M, Fisher B, Taphoorn MJ, et al Radiotherapy plus concomitant and adjuvant temozolomide for glioblastoma. N Engl J Med. 2005;352: 987–996. 10.1056/NEJMoa043330 15758009

[2] Stupp R, Hegi ME, Mason WP, van den Bent MJ, Taphoorn MJ, Janzer RC, et al Effects of radiotherapy with concomitant and adjuvant temozolomide versus radiotherapy alone on survival in glioblastoma in a randomised phase III study: 5-year analysis of the EORTC-NCIC trial. Lancet Oncol. 2009;10: 459–466. 10.1016/S1470-2045(09)70025-7 19269895

[3] Cooper MA, Fehniger TA, Caligiuri MA. The biology of human natural killer-cell subsets. Trends Immunol. 2001;22: 633–640. 11698225 10.1016/s1471-4906(01)02060-9

[4] Li Y, Sun R. Tumor immunotherapy: New aspects of natural killer cells. Chin J Cancer Res. 2018;30: 173–196. 10.21147/j.issn.1000-9604.2018.02.02 29861604 PMC5953955

[5] Caligiuri MA. Human natural killer cells. Blood. 2008;112: 461–469. 10.1182/blood-2007-09-077438 18650461 PMC2481557

[6] Uppendahl LD, Dahl CM, Miller JS, Felices M, Geller MA. Natural killer cell-based immunotherapy in gynecologic malignancy: A review. Front Immunol. 2017;8: 1825 10.3389/fimmu.2017.01825 29354116 PMC5760535

[7] Paul S, Lal G. The molecular mechanism of natural killer cells function and its importance in cancer immunotherapy. Front Immunol. 2017;8: 1124 10.3389/fimmu.2017.01124 28955340 PMC5601256

[8] Lopez JA, Susanto O, Jenkins MR, Lukoyanova N, Sutton VR, Law RH, et al Perforin forms transient pores on the target cell plasma membrane to facilitate rapid access of granzymes during killer cell attack. Blood. 2013;121: 2659–2668. 10.1182/blood-2012-07-446146 23377437

[9] Li J, Figueira SK, Vrazo AC, Binkowski BF, Butler BL, Tabata Y, et al Real-time detection of CTL function reveals distinct patterns of caspase activation mediated by Fas versus granzyme B. J Immunol. 2014;193: 519–528. 10.4049/jimmunol.1301668 24928990 PMC4107314

[10] Lanier LL. NK cell recognition. Annu Rev Immunol. 2005;23: 225–274. 10.1146/annurev.immunol.23.021704.115526 15771571

[11] Morvan MG, Lanier LL. NK cells and cancer: you can teach innate cells new tricks. Nat Rev Cancer. 2016;16: 7–19. 10.1038/nrc.2015.5 26694935

[12] Jung TY, Choi YD, Kim YH, Lee JJ, Kim HS, Kim JS, et al Immunological characterization of glioblastoma cells for immunotherapy. Anticancer Res. 2013;33(6): 2525–2533. 23749904

[13] Champsaur M, Lanier LL. Effect of NKG2D ligand expression on host immune responses. Immunol Rev. 2010;235: 267–285. 10.1111/j.0105-2896.2010.00893.x 20536569 PMC2885032

[14] Braud VM, Allan DS, O'callaghan CA, Söderström K, D'andrea A, Ogg GS, et al HLA-E binds to natural killer cell receptors CD94/NKG2A, B and C. Nature. 1998;391: 795–799. 10.1038/35869 9486650

[15] Winter CC, Gumperz JE, Parham P, Long EO, Wagtmann N. Direct binding and functional transfer of NK cell inhibitory receptors reveal novel patterns of HLA-C allotype recognition. J Immunol. 1998;16: 571–577. 9670929

[16] Cheng M, Chen Y, Xiao W, Sun R, Tian Z. NK cell-based immunotherapy for malignant diseases. Cell Mol Immunol. 2013;10: 230–252. 10.1038/cmi.2013.10 23604045 PMC4076738

[17] Guillerey C, Huntington ND, Smyth MJ. Targeting natural killer cells in cancer immunotherapy. Nat Immunol. 2016;17: 1025–1036. 10.1038/ni.3518 27540992

[18] Ishikawa E, Takano S, Ohno T, Tsuboi K. Adoptive cell transfer therapy for malignant gliomas. Adv Exp Med Biol. 2012;746: 109–120. 10.1007/978-1-4614-3146-6_9 22639163

[19] Dillman RO, Duma CM, Ellis RA, Cornforth AN, Schiltz PM, Sharp SL, et al Intralesional lymphokine-activated killer cells as adjuvant therapy for primary glioblastoma. J Immunother. 2009;32: 914–319. 10.1097/CJI.0b013e3181b2910f 19816190

[20] Ishikawa E, Tsuboi K, Saijo K, Harada H, Takano S, Nose T, et al Autologous natural killer cell therapy for human recurrent malignant glioma. Anticancer Res. 2004;24: 1861–1871. 15274367

[21] Ghiringhelli F, Ménard C, Martin F, Zitvogel L. The role of regulatory T cells in the control of natural killer cells: relevance during tumor progression. Immunol Rev. 2006;214: 229–238. 10.1111/j.1600-065X.2006.00445.x 17100888

[22] Lichtenfels R, Biddison WE, Schulz H, Vogt AB, Martin R. CARE-LASS (calcein-release-assay), an improved fluorescence-based test system to measure cytotoxic T lymphocyte activity. J Immunol Methods. 1994;172: 227–239. 7518485 10.1016/0022-1759(94)90110-4

[23] Cornelissen M, Philippé J, De Sitter S, De Ridder L. Annexin V expression in apoptotic peripheral blood lymphocytes: an electron microscopic evaluation. Apoptosis. 2002;7: 41–47. 11773704 10.1023/a:1013560828090

[24] Nakazawa T, Nakamura M, Matsuda R, Nishimura F, Park YS, Motoyama Y, et al Antitumor effects of minodronate, a third-generation nitrogen-containing bisphosphonate, in synergy with gammadeltaT cells in human glioblastoma *in vitro* and *in vivo*. J Neurooncol. 2016;129: 231–241. 10.1007/s11060-016-2186-x 27393349

[25] Vivier E, Tomasello E, Baratin M, Walzer T, Ugolini S. Functions of natural killer cells. Nat Immunol. 2008;9: 503–510. 10.1038/ni1582 18425107

[26] Yang I, Han SJ, Sughrue ME, Tihan T, Parsa AT. Immune cell infiltrate differences in pilocytic astrocytoma and glioblastoma: evidence of distinct immunological microenvironments that reflect tumor biology. J Neurosurg. 2011;115: 505–511. 10.3171/2011.4.JNS101172 21663411

[27] Kmiecik J, Poli A, Brons NH, Waha A, Eide GE, Enger PØ, et al Elevated CD3+ and CD8+ tumor-infiltrating immune cells correlate with prolonged survival in glioblastoma patients despite integrated immunosuppressive mechanisms in the tumor microenvironment and at the systemic level. J Neuroimmunol. 2013;264: 71–83. 10.1016/j.jneuroim.2013.08.013 24045166

[28] Kmiecik J, Zimmer J, Chekenya M. Natural killer cells in intracranial neoplasms: presence and therapeutic efficacy against brain tumours. J Neurooncol. 2014;116: 1–9. 10.1007/s11060-013-1265-5 24085644 PMC3889498

[29] Bielamowicz KJ, Khawja S, Ahmed N. Ahmed, Adoptive cell therapies for glioblastoma. Front Oncol. 2013;3: 275 10.3389/fonc.2013.00275 24273748 PMC3823029

[30] Granzin M, Soltenborn S, Müller S, Kollet J, Berg M, Cerwenka A, et al Fully automated expansion and activation of clinical-grade natural killer cells for adoptive immunotherapy. Cytotherapy. 2015;17: 621–632. 10.1016/j.jcyt.2015.03.611 25881519 PMC8725994

[31] Lee HR, Son CH, Koh EK, Bae JH, Kang CD, Yang K, et al Expansion of cytotoxic natural killer cells using irradiated autologous peripheral blood mononuclear cells and anti-CD16 antibody. Sci Rep. 2017;7: 11075 10.1038/s41598-017-09259-1 28894091 PMC5593981

[32] Sakamoto N, Ishikawa T, Kokura S, Okayama T, Oka K, Ideno M, et al Phase I clinical trial of autologous NK cell therapy using novel expansion method in patients with advanced digestive cancer. J Transl Med. 2015;13: 277 10.1186/s12967-015-0632-8 26303618 PMC4548900

[33] Koehl U, Esser R, Zimmermann S, Tonn T, Kotchetkov R, Bartling T, et al *Ex vivo* expansion of highly purified NK cells for immunotherapy after haploidentical stem cell transplantation in children. Klin Padiatr. 2005;217: 345–350. 10.1055/s-2005-872520 16307421

[34] Yu B, Wang J, He C, Wang W, Tang J, Zheng R, et al Cytokine-induced killer cell therapy for modulating regulatory T cells in patients with non-small cell lung cancer. Exp Ther Med. 2017;14: 831–840. 10.3892/etm.2017.4562 28673007 PMC5488714

[35] Vitale M, Cantoni C, Pietra G, Mingari MC, Moretta L. Effect of tumor cells and tumor microenvironment on NK-cell function. Eur J Immunol. 2014;44: 1582–1592. 10.1002/eji.201344272 24777896

[36] Raulet DH, Held W. Natural killer cell receptors: the offs and ons of NK cell recognition. Cell. 1995;82: 697–700. 7671299 10.1016/0092-8674(95)90466-2

[37] Vitale M, Cantoni C, Pietra G, Mingari MC, Moretta L. Natural killer cell signaling pathways. Science. 2004;306: 1517–1519. 10.1126/science.1103478 15567854

[38] Sawamura Y, Diserens AC, de Tribolet N. *In vitro* prostaglandin E2 production by glioblastoma cells and its effect on interleukin-2 activation of oncolytic lymphocytes. J Neurooncol. 1990;9: 125–130. 2175768 10.1007/BF02427832

[39] Lee HW, Singh TD, Lee SW, Ha JH, Rehemtulla A, Ahn BC, et al Evaluation of therapeutic effects of natural killer (NK) cell-based immunotherapy in mice using *in vivo* apoptosis bioimaging with a caspase-3 sensor. FASEB J. 2014;28: 2932–2941. 10.1096/fj.13-243014 24736413

[40] Björklund AT, Carlsten M, Sohlberg E, Liu LL, Clancy T, Karimi M, et al Complete remission with reduction of high-risk clones following haploidentical NK-cell therapy against MDS and AML. Clin Cancer Res. 2018;24: 1834–1844. 10.1158/1078-0432.CCR-17-3196 29444931

[41] Burga RA, Nguyen T, Zulovich J, Madonna S, Ylisastigui L, Fernandes R, et al Improving efficacy of cancer immunotherapy by genetic modification of natural killer cells. Cytotherapy. 2016;18: 1410–1421. 10.1016/j.jcyt.2016.05.018 27421740

